# Importance of Kupffer Cells in the Development of Acute Liver Injuries in Mice

**DOI:** 10.3390/ijms15057711

**Published:** 2014-05-05

**Authors:** Hiroko Tsutsui, Shuhei Nishiguchi

**Affiliations:** 1 Department of Microbiology, Hyogo College of Medicine, 1-1 Mukogawa-cho, Nishinomiya 663-8501, Japan; 2 Department of Internal Medicine, Hyogo College of Medicine, 1-1 Mukogawa-cho, Nishinomiya 663-8501, Japan; E-Mail: nishiguc@hyo-med.ac.jp

**Keywords:** *Propionibacterium acnes*, lipopolysaccharide (LPS), Toll-like receptor (TLR), inflammasome, interleukin-18 (IL-18), Fas, concanavalin A (Con A), acetaminophen (APAP), tissue factor, procoagulant and anticoagulant pathways

## Abstract

Kupffer cells reside within the liver sinusoid and serve as gatekeepers. They produce pro- and anti-inflammatory cytokines and other biologically important molecules upon the engagement of pattern recognition receptors such as Toll-like receptors. Kupffer cell-ablated mice established by *in vivo* treatment with clodronate liposomes have revealed many important features of Kupffer cells. In this paper, we review the importance of Kupffer cells in murine acute liver injuries and focus on the following two models: lipopolysaccharide (LPS)-induced liver injury, which is induced by priming with *Propionibacterium acne*s and subsequent challenge with LPS, and hypercoagulability-mediated acute liver failure such as that in concanavalin A (Con A)-induced hepatitis. Kupffer cells are required for LPS sensitization induced by *P. acnes* and are a major cellular source of interleukin-18, which induces acute liver injury following LPS challenge. Kupffer cells contribute to Con A-induced acute liver failure by initiating pathogenic, intrasinusoidal thrombosis in collaboration with sinusoidal endothelial cells. The mechanisms underlying these models may shed light on human liver injuries induced by various etiologies such as viral infection and/or abnormal metabolism.

## 1. Introduction

**Figure 1.**
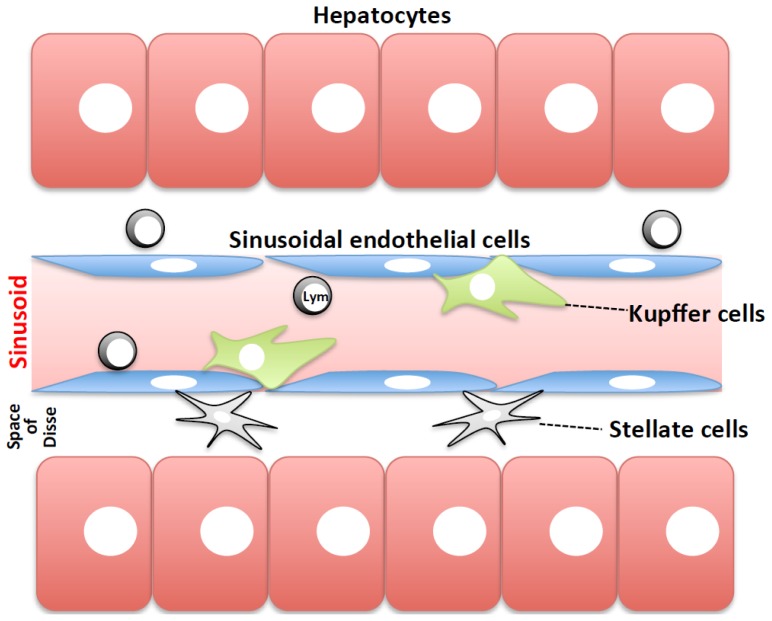
Schematic structure of the liver. Hepatic sinusoids are composed of sinusoidal endothelial and Kupffer cells. Blood circulates within the sinusoids in the liver. Hepatocytes, or liver parenchymal cells are localized to the space of Disse, distant from the sinusoid. Natural killer (NK) and invariant NK T (NKT) cells are abundant in the liver. Lym: lymphocytes, including NK cells, NKT cells, and other lymphocytes.

The liver is anatomically connected with the intestinal system by the portal vein. The nutrients absorbed from the digestive system are carried into the mesenteric veins, which converge into the portal vein. Thus, the liver serves as an industrial complex in which the parenchymal cells maintain normal metabolism by utilizing the nutrients to fine-tune the catabolic and anabolic pathways. To efficiently extract nutrients from the food we eat, our digestive system relies on indigenous, commensal resident bacteria. Therefore, the gut-derived bacteria, or their products, can incidentally reach the liver [1]. The liver consists of parenchymal (hepatocytes) and non-parenchymal cells that are primarily divided into three types as follows: Kupffer cells, which are tissue macrophages localized within the liver sinusoid and serve as nurses for homeostatic liver regeneration as well as gatekeepers [2,3]; sinusoidal endothelial cells; and stellate cells, which were originally designated “Ito cells”, that exist within the space of Disse, apart from the sinusoid [1,4] (Figure 1). Lymphocytes reside in the liver as well. Natural killer (NK) cells, which were previously histologically referred to as “pit cells” [5,6], and invariant NK T (NKT) cells, which express NK cell markers and the limited T-cell antigen receptor (TCR) repertoire in Vα and Vβ chains and have the potential to promptly produce large amounts of pro- and anti-inflammatory cytokines upon TCR engagement [7,8], are exceptionally abundant in the liver compared with other organs. These lymphocytes are localized in both the sinusoid and space of Disse [5,9] (Figure 1). Among the cell types, Kupffer, NK, and invariant NKT cells are hematopoietic cells, whereas the other cell types are non-hematopoietic cells.

In mice, two different types of precursor cells differentiate into macrophages [10–12]. One is derived from the yolk sac and the other from hematopoietic stem cells (HSCs). Macrophages develop in the yolk sac in the early embryonic stage. In the later stage, HSCs appear in the aorta-gonad-mesonephros region and migrate to the fetal liver, where they expand and differentiate into various cell types, including macrophages. Recently, Kupffer cells were verified to be derived from the yolk sac, not HSCs [13]. The difference in cell lineages might reflect the features of the two types of macrophages. Conventional HSC-derived hematopoietic cells such as T cells, B cells, and macrophages are radiosensitive, whereas Kupffer cells are radioresistant [14]. It is noteworthy that regular bone marrow transfer cannot reconstitute Kupffer cells. If host mice sequentially receive irradiation and then transplantation of donor bone marrow cells, T cells, B cells, and many other hematopoietic cell types, but not Kupffer cells, are thoroughly reconstituted with donor-derived cells [14]. For successful reconstitution of Kupffer cells, ablation of Kupffer cells prior to host irradiation is required [14,15].

In this paper, we review the importance of Kupffer cells in the development of acute liver injuries with various etiologies, particularly focusing on recent findings in mice.

## 2. Endotoxin-Induced Liver Injury

Mice are somewhat resistant to lipopolysaccharide (LPS), a cell wall component of gram-negative bacteria. The 50% lethal dose (LD_50_) of LPS in non-treated naive mice is approximately 50 mg/kg. However, systemic administration of heat-killed *Propionibacterium acnes* (previously named *Corynebacterium parvum*), an indigenous bacterium in skin, render mice highly susceptible to LPS [16,17]. The LD_50_ of LPS in *P. acnes*-primed mice is <50 μg/kg. The morbidity and mortality of *P. acnes*-primed mice after challenge with a subclinical dose of LPS were demonstrated. After the LPS challenge, the *P. acnes*-primed mice developed proinflammatory cytokinemia, systemic hypercoagulation, and hypothermia, followed by a high mortality [18]. All these signs were observed in severe septic patients [19,20]. The surviving mice developed acute liver injury later [21,22]. In contrast, the non-treated naive mice displayed none of the symptoms and signs after challenge with the same LPS dose. Thus, systemic treatment with heat-killed *P. acnes* prominently enhanced the sensitivity of the mice to LPS. The mechanisms underlying the *P. acnes*-induced sensitization to LPS and the morbidity and mortality after the subsequent LPS challenge differed. Therefore, we will introduce each mechanism separately.

### 2.1. P. acnes Induction of LPS Sensitization

Treatment of mice with heat-killed *P. acnes* induced the formation of dense granulomas in the liver that largely consisted of F4/80^+^ macrophages [21,23]. To address the mechanism of this hepatic granuloma formation, we investigated which cells ingested heat-killed *P. acnes* via fluorescence labeling of the bacteria. We found that labeled bacteria were promptly ingested predominantly by F4/80^+^ Kupffer cells in the liver but by F4/80^−^ cells in the spleen [22,23]. Intriguingly, hepatic granulomas grew and expanded around the *P. acnes*-phagocytized Kupffer cells [22], prompting us to propose the possibility that Kupffer cells are necessary for the development of hepatic granulomas. To test this, we depleted Kupffer cells by intravenous injection of clodronate liposome, which selectively eliminates macrophages, including Kupffer cells and dendritic cells [15]. Mice pretreated with clodronate liposome, which we designated as “Kupffer cell-ablated mice”, did not exhibit hepatic granuloma formation after treatment with heat-killed *P. acnes*. Furthermore, in contrast to Kupffer cell-sufficient mice, the Kupffer cell-ablated mice remained resistant to LPS-induced mortality and liver injury, even after receiving heat-killed *P. acnes* [18,23]. Thus, Kupffer cells, and/or presumably other clodronate liposome-sensitive cells, are primarily required for *P. acnes*-induced LPS sensitization, both systemically and locally, via the induction of hepatic granuloma formation.

**Figure 2.**
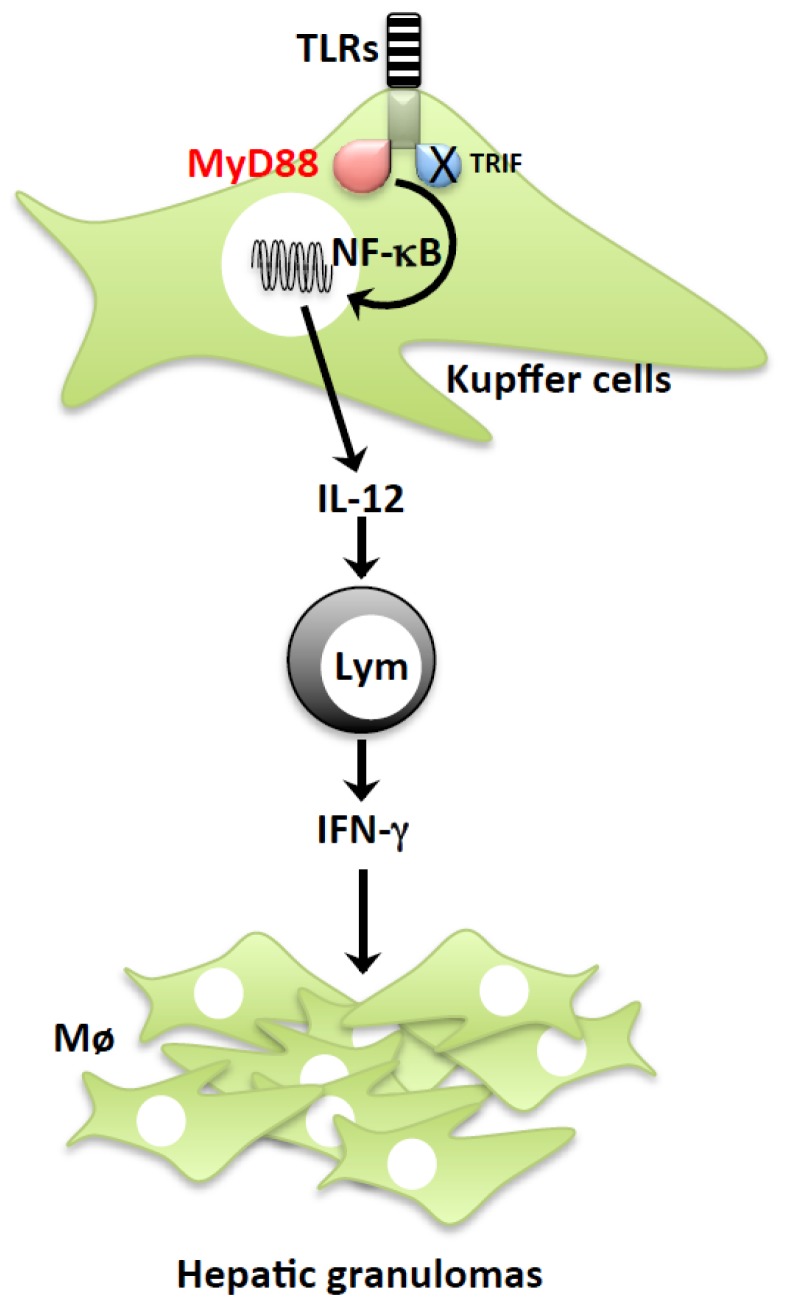
The MyD88-IL-12-IFN-γ axis is important for *P. acnes-*induced LPS sensitization. After systemic treatment with heat-killed *P. acnes*, the Kupffer cells ingested the bacteria and activated the TLR-MyD88-NF-κB pathway, thereby producing IL-12. In response to the IL-12 expression, lymphocytes produced IFN-γ, which eventually activated dense granulomas consisting of macrophages in the liver.

Myeloid differentiation factor 88 (MyD88) is a common adapter molecule for the signal pathways mediated by Toll-like receptor (TLR) family members, with the exception of TLR3 [24]. TLRs are innate immune signal receptors that sense extracellular and endosomal, but not cytoplasmic, danger molecules. As they cannot invade the cytosol of cells after being ingested by Kupffer cells, heat-killed *P. acnes* is thought to be recognized by extracellular/endosomal sensor TLRs. Therefore, it is plausible that *Myd88*^−/−^ mice do not develop hepatic granulomas after *P. acnes* treatment [23,25]. Indeed, mice deficient in TLR9, which utilizes MyD88 for signaling, are somewhat resistant to *P. acnes*-induced hepatic granulomatous changes [26], suggesting the importance of TLR9-MyD88 pathway for the development of *P. acnes*-induced hepatic granuloma formation. The TLR/MyD88-mediated pathway activates nuclear factor-kappa B (NF-κB) to induce gene expression of various molecules, including interleukin (IL) 12. It is conceivable that the introduction of NF-κB decoy oligodeoxynucleotides in the liver protects against *P. acnes*-induced hepatic granuloma formation, and the resultant morbidity and mortality, after subsequent challenge with a subclinical dose of LPS [27]. *Il12*^−/−^ mice are also resistant to *P. acnes* treatment in terms of hepatic granuloma formation and the acquisition of LPS sensitization [18,28]. As IL-12 is a potent interferon (IFN)-γ-inducing factor, IFN-γ expression seems to also be involved in this phenomenon. Indeed, *Ifn-γ*^−/−^ mice do not show these host responses to *P. acnes* at all [18,29]. Furthermore, *P. acnes* treatment renders wild-type mice highly susceptible to tumor necrosis factor-α (TNF), in terms of morbidity and mortality, similar to *P. acnes*/LPS-induced illness [18]. Intriguingly, *P. acnes*-induced sensitization to TNF also depends on IL-12 and IFN-γ expression [18]. Collectively, the TLR-MyD88-IL-12-IFN-γ axis is required for the development of *P. acnes*-induced hepatic granuloma formation and the resultant sensitization to LPS and TNF (Figure 2).

### 2.2. LPS-Induced Liver Injury

The importance of IL-18 for the development of *P. acnes*/LPS-induced liver injury was reported soon after the identification of IL-18 as a potent IFN-γ-inducing factor [30]. Administration of neutralizing anti-IL-18 antibodies just before LPS treatment rescued *P. acnes*-primed mice from the liver injury induced by LPS challenge. Consistently, *P. acnes*-primed *Il18*^−/−^ mice were resistant to LPS-induced liver injury concomitant with the impairment in IFN-γ induction. This suggests that IL-18, like IL-12, is also involved in the *P. acnes* induction of LPS sensitization by inducing IFN-γ expression. However, in contrast with *Il12*^−/−^ mice, *Il18*^−/−^ mice exhibit normal susceptibility to *P. acnes* treatment in terms of hepatic granuloma development and LPS hypersensitiveness [28].

**Figure 3.**
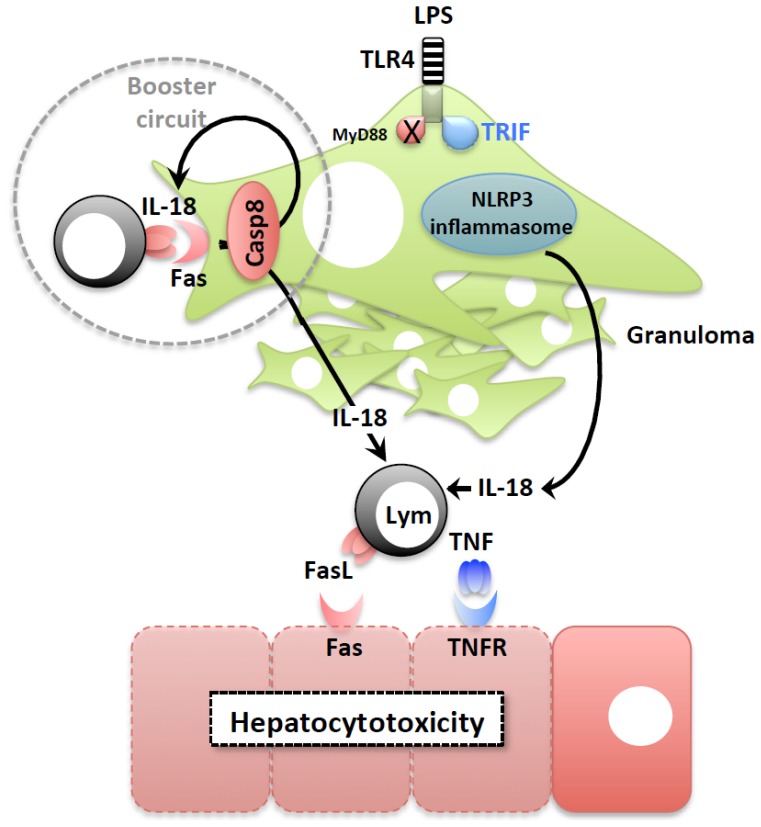
TLR4-TRIF-NLRP3 inflammasome-IL-18 axis for lipopolysaccharide (LPS)-induced liver injury. After LPS challenge, the TLR4-TRIF-mediated signal is transduced in the *P. acnes*-elicited hepatic macrophages to activate the NLRP3 inflammasome, in which precursor caspase-1 becomes enzymatically active caspase-1. Caspase-1 then cleaves precursor IL-18 to secrete mature IL-18. IL-18 activates lymphocytes such as neutral killer cells to express Fas ligand (FasL) and produce tumor necrosis factor-α (TNF). Both FasL and TNF induce apoptotic hepatocyte cell death. *P. acnes*-elicited hepatic macrophages express Fas. Upon FasL stimulation, *P. acnes*-elicited hepatic macrophages produce mature IL-18 in a caspase-8-dependent manner. Thus, IL-18-induced FasL expression and Fas-mediated caspase-8-dependent IL-18 release serve as the booster circuit (shown by the dotted circle), which may accelerate the development of liver injury.

IL-18 is a member of the IL-1 family and is produced as a biologically inactive precursor [30,31]. Cleavage of precursor IL-18 is required for its activation and secretion. Caspase (Casp)-1 is the primary intracellular enzyme that processes precursor IL-18 and IL-1β into their mature forms [32]. *Casp1*^−/−^ mice, which were demonstrated strictly to be *Casp1*^−/−^*Casp11*^−/−^ mice owing to the caspase-11 deficiency in their ancestor embryonic stem cells [33], display the same phenotype as that seen in *Il18*^−/−^ mice [23]. Caspase-1 is also produced as a biologically inactive zymogen. Caspase-1 activation occurs in association with the inflammasome, a cytoplasmic, large, protein complex formed after cell activation with the appropriate stimuli [34]. Inflammasomes can be separated into at least four types, distinguished by the use of different cytoplasmic sensors, namely NACHT, leucine-rich repeat (LRR), and pyrin domain-containing protein (NLRP)-1; NLRP3; NACHT, LRR, and caspase recruitment domain (CARD) domain-containing protein (NLRC)-4; and absent in melanoma 2 (AIM2). Three of the four sensors, namely NLRP1, NLRP3, and NLRC4, belong to the NOD-like receptor (NLR) family, and AIM2 is a DNA sensor outside the NLR family [34,35]. We verified that the NLRP3 inflammasome is required for caspase-1 activation in Kupffer cells following stimulation with LPS [23]. Kupffer cells that lack NLRP3 or the gene encoding apoptosis-associated speck-like protein containing a carboxy-terminal CARD (ASC), an adaptor molecule of caspase-1 activation [36], cannot exhibit caspase-1 activation or IL-18/IL-1β maturation upon LPS stimulation. TLR4, a cell surface receptor for LPS, is critical for LPS-induced caspase-1 activation [23]. Furthermore, LPS-activated TLR4-mediated caspase-1 activation is mediated by Toll/IL-1 receptor domain-containing adaptor inducing IFN-β (TRIF), but not MyD88, in Kupffer cells [23]. Thus, upon TLR4 engagement, TRIF-mediated signaling activates the NLRP3 inflammasome to release biologically active IL-18 and IL-1β, at least from Kupffer cells (Figure 3). However, the molecular process that links the TLR4/TRIF signal to the activation of the NLRP3 inflammasome is not fully understood.

Intriguingly, IL-18 cannot directly kill hepatocytes. Primary cultured hepatocytes do not show necrotic or apoptotic cell death after incubation with recombinant IL-18 (rIL-18). How does IL-18 contribute to *P. acnes*/LPS-induced liver cell death? IL-18 has the capacity to induce molecules that can in turn induce hepatocyte cell death. IL-18 can induce Fas ligand (FasL) expression in Natural killer (NK) cells [37]. As hepatocytes constitutively express Fas, a potent cell death signal receptor [38], the contribution of IL-18 to liver injury via induction of FasL becomes conceivable. Furthermore, IL-18 is capable of inducing TNF, another powerful hepatocytocidal cytokine [39], both directly and indirectly [21,40,41]. Thus, after LPS challenge, the release of IL-18 via activation of the TLR4/TRIF-NLRP3 inflammasome axis is important for the development of liver injury via induction of hepatocytocidal factors (Figure 3).

### 2.3. IL-18 Induction of FasL Accelerates the Development of Liver Injury

Macrophages do not express Fas in the resting state. However, liver macrophages from *P. acnes*-primed mice express Fas molecules on their surfaces. As shown previously, FasL-expressing cells can kill Fas-expressing hepatocytes, but not hepatic macrophages, although the mechanism is poorly defined [38,42,43]. Upon FasL stimulation, Fas-expressing macrophages release mature IL-18 and IL-1β [42,43]. This suggests that Fas-mediated signaling might activate inflammasomes. However, the FasL-induced release of IL-18 and IL-1β does not require caspase-1, NLRP3, or NLRC4 [42,43]. Very recently, caspase-8, but not caspase-1, caspase-9, or caspase-11, was demonstrated to be critical for Fas-mediated IL-18 maturation [44,45]. As mentioned above, IL-18 can induce FasL expression by NK cells [37]. This strongly suggests that the IL-18-induced expression of FasL might promote liver injury via the acceleration of the release of abundant amounts of mature IL-18. In fact, administration of rFasL- or FasL-expressing NK cells caused acute liver injury concomitant with elevated serum levels of IL-18 in *P. acnes*-primed mice, but not in naive mice [42]. Furthermore, intravenous injection of rIL-18 caused liver injury, partly dependent on endogenous Fas/FasL signaling, in *P. acnes*-primed mice [42]. Thus, IL-18 can accelerate the release of mature IL-18 via the induction of FasL expression and activation of Fas-mediated caspase-8 signaling. Once IL-18 is released, the “IL-18 booster circuit” could burst, which could eventually result in the explosive expansion of the liver injury [21,42] (Figure 3).

## 3. Hypercoagulation-Associated Acute Severe Hepatitis

Most of the proteins involved in the procoagulant and anticoagulant pathways are synthesized in the liver, which may imply that patients with liver injuries undergo changes in their hemostatic system that make them susceptible to coagulopathy-prone conditions [46]. However, we now know that patients with chronic liver injuries exemplified by liver cirrhosis show “rebalanced hemostatic status” due to a reduction in both procoagulant and anticoagulant mechanisms or rather a predisposition to the hypercoagulability state [47–49]. Very recently, hemostatic alterations in acute liver failure were demonstrated to be similar to those in chronic liver diseases [50–52]. This is also the case for mouse models of acute liver failure such as those induced by concanavalin A (Con A) and acetaminophen (APAP) administration. In both models, hypercoagulability contributes to the development of severe liver failure, but through different cellular and molecular mechanisms.

### 3.1. Con A-Induced Acute Liver Injury

Con A is a jack bean-derived lectin with powerful T-cell mitogenic activity. Intravenous injection of Con A induces acute liver failure in mice [53]. It has been well established that T cells, particularly CD4^+^ cells, play an essential role in the development of Con A-induced hepatitis [53]. Considering that neutralization or gene depletion of IFN-γ or TNF prevents mice from developing Con A hepatitis, IFN-γ and TNF were believed to serve as critical initiators of inflammation in this type of liver injury. Recently, we clearly demonstrated that IFN-γ and TNF were responsible for Con A hepatitis; however, this occurred by the induction of intrasinusoidal prothrombosis. Microthrombi consisting of red blood cells, platelets, and fibrin deposits were observed under electron microscopy of the liver of Con A-challenged mice [54]. Inhibition of the coagulation cascade can potently rescue mice from Con A hepatitis without impairment in the induction of IFN-γ and TNF [54]. Very recently, we identified the cellular and molecular mechanisms for the development of Con A-induced acute prothrombotic liver failure [55].

#### 3.1.1. Importance of IFN-γ/STAT1 in the Development of Procoagulant Hepatitis

**Figure 4.**
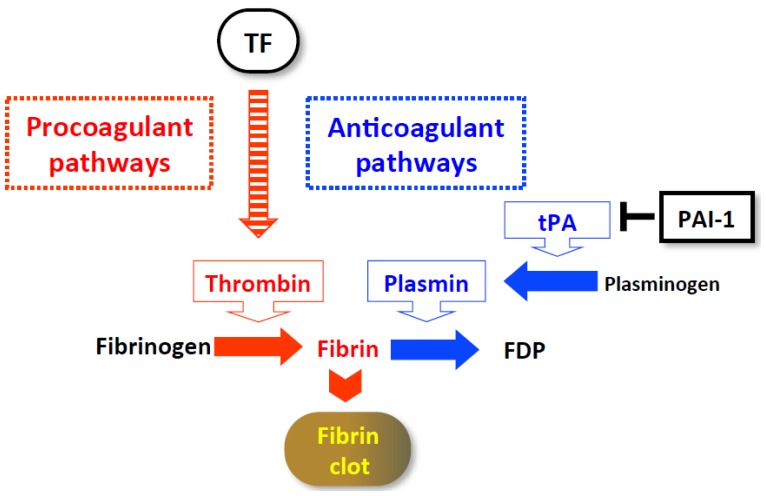
Hemostatic system. After factor VII binding to tissue factor (TF), the activation of the coagulation cascade begins, leading to the conversion of fibrinogen into fibrin by thrombin and the activation of platelets. Subsequently, insoluble fibrin clots are formed. The coagulation cascade is tightly regulated by the anticoagulant pathway. Tissue plasminogen activator (tPA) cleaves plasminogen, converting it to biologically active plasmin. Plasmin then degrades fibrin into soluble fibrin-degraded products (FDP) to protect against thrombosis. Plasminogen activator inhibitor type 1 (PAI-1) inhibits tPA activity. Thus, PAI-1 participates in coagulation by inhibiting the anticoagulant pathway.

**Figure 5.**
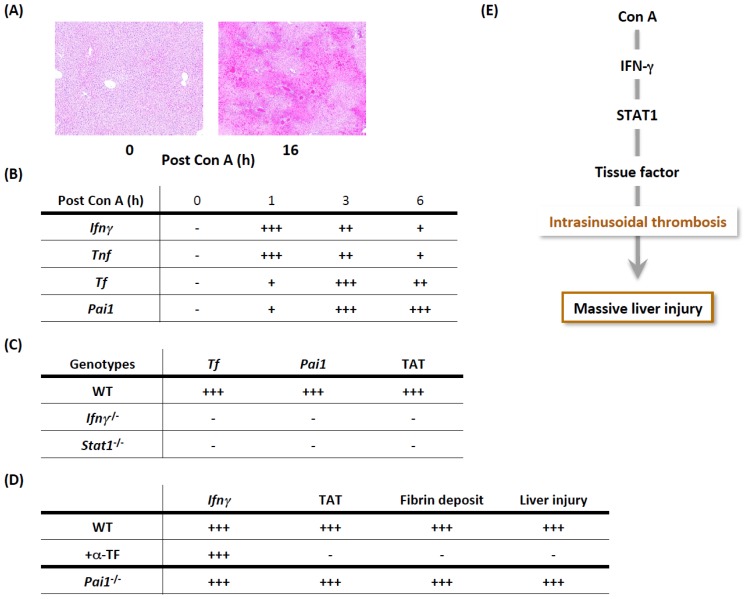
IFN-γ/TNF-mediated TF induction contributes to procoagulant liver injury. (**A**) After Con A challenge, massive liver injury developed; (**B**) The liver showed prompt expressions of *Ifnγ* and *Tnf*, followed by those of *Tf* (Tissue factor) and *Pai1* (Plasminogen activator inhibitor type 1); (**C**) Wild-type (WT) mice exhibited induction of both *Tf* and *Pai1* in the liver and elevated plasma levels of thrombin-anti-thrombin III complex (TAT), which is an excellent indicator of labile thrombin. In contrast, *Ifnγ*^−/−^ mice and mice deficient in transcription factor of IFN-γ signaling, Signal Transducers and Activator of Transcription (STAT)-1, did not show any of these changes after Con A challenge, indicating that the IFN-γ-STAT1 signaling is prerequisite for these responses; (**D**) *Pai1*^−/−^ mice exhibited the hepatic induction of *Ifnγ*, plasma elevation of TAT levels, hepatic fibrin deposition, and liver injury comparably as did WT mice. In contrast, pretreatment of WT mice with neutralizing anti-TF monoclonal antibody (α-TF) inhibited all the responses except for induction of *Ifnγ*. Thus, TF but not PAI-1 is necessarily required for the development of prothrombotic hepatitis, and IFN-γ signaling does not participate in this liver injury unless the coagulation cascade is activated; and (**E**) These results indicate that Con A activates the STAT1-mediated IFN-γ pathway to induce TF expression, which eventually results in massive liver necrosis concomitant with dense intrasinusoidal thrombosis.

Tissue factor (TF) is a primary activator of procoagulant pathways [56–58], which results in fibrin formation and platelet activation (Figure 4). After Con A challenge, massive liver injury develops and is accompanied by dense, intrasinusoidal fibrin deposition [55] (Figure 5A). Plasma levels of the thrombin-antithrombin III complex (TAT), an excellent indicator of thrombin, are exceptionally elevated, clearly indicating that systemic hypercoagulation develops as well. After Con A challenge, hepatic expression of *Ifnγ* and *Tnf* are promptly induced, followed by hepatic induction of *Tf* (Figure 5B). Plasminogen activator inhibitor type 1 (PAI-1) promotes coagulation by potently inhibiting anticoagulant pathways [59,60] (Figure 4). Like *Tf*, hepatic *Pai1* induction follows *Ifnγ* and *Tnf* induction (Figure 5B). Signal transducer and activator of transcription (STAT)-1, a transcription factor for IFN-γ signaling, is necessary for the hepatic induction of *Tnf* and *Pai1* (Figure 5C), indicating the importance of IFN-γ/STAT1. Notably, a neutralizing anti-TF monoclonal antibody protects against liver injury and concomitantly inhibits intrasinusoidal fibrin deposition. In sharp contrast, *Pai1*^−/−^ mice are normally susceptible to Con A hepatitis (Figure 5D). Intriguingly, the neutralization of TF does not affect hepatic *Ifnγ* induction (Figure 5D), clearly indicating that IFN-γ contributes to Con A hepatitis primarily by initiating TF, but only slightly by activating inflammatory responses. Accordingly, TF, but not PAI-1, is critical for the development of thrombotic liver injury. Thus, IFN-γ-mediated STAT1 signaling induces TF expression, leading to the development of massive prothrombotic liver injury (Figure 5E).

#### 3.1.2. Cellular Mechanisms of Prothrombotic Hepatitis

**Figure 6.**
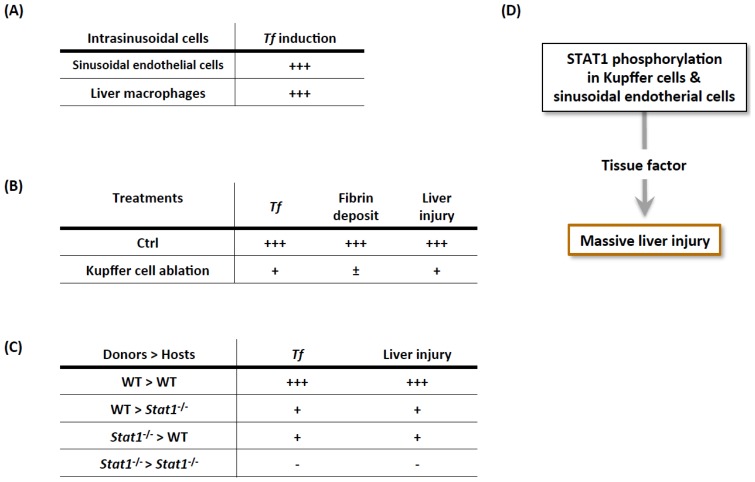
Both hepatic macrophages and sinusoidal endothelial cells are equally required for the development of procoagulant hepatitis via STAT1 activation of TF expression. (**A**) After Con A challenge, both the sinusoidal endothelial cells and liver macrophages in the WT mice expressed significantly higher levels of TF than those from naive WT mice; (**B**) The Kupffer cell-ablated mice displayed partially impaired induction of hepatic TF, hepatic fibrin deposition, and liver injury; (**C**) The WT mice that received WT bone marrow cells were susceptible to Con A-induced pathological changes. The *Stat1*^−/−^ mice that received *Stat1*^−/−^ bone marrow cells were completely resistant to Con A. The WT mice that received *Stat1*^−/−^ bone marrow cells and the *Stat1*^−/−^ mice that received WT bone marrow cells almost equally evaded Con A-induced alterations; and (**D**) STAT1 signaling in both the Kupffer and sinusoidal endothelial cells equally contributed to the hepatic induction of TF, eventually leading to prothrombotic liver injury.

The sinusoid consists of two types of cells that have the potential to express TF: Kupffer cells and sinusoidal endothelial cells (Figure 1). Compared with control mice, both sinusoidal endothelial cells and Kupffer cells isolated from Con A-treated mice exhibited obvious induction of *Tf* expression, suggesting that both cell types might contribute to the initiation of the coagulation cascade in the liver (Figure 6A). To clarify the roles of Kupffer cells, we generated Kupffer cell-ablated mice by injecting clodronate liposome, as described earlier [15]. The Kupffer cell-ablated mice showed a reduction in *Tf* induction and reduced liver injury relative to mice treated with a control PBS liposome, indicating that Kupffer cells are responsible for prothrombotic liver injury via the expression of *Tf* (Figure 6B). To further examine which cell type, either Kupffer or sinusoidal endothelial cell, is primarily responsible for hepatic microthrombosis, we generated various chimeric mice. Kupffer cell-ablated host mice were regularly irradiated, and then they received donor bone marrow cells. WT mice reconstituted with WT hematopoietic cells showed hepatic *Tf* induction and developed liver injury after Con A challenge (WT > WT). In contrast, the *Stat1*^−/−^ mice reconstituted with *Stat1*^−/−^ hematopoietic cells were resistant to Con A treatment, as seen with the *Stat1*^−/−^ mice (*Stat1*^−/−^ > *Stat1*^−/−^; Figure 6C). The WT mice reconstituted with *Stat1*^−/−^ hematopoietic cells (*Stat1*^−/−^ > WT) and the converse chimera, the *Stat1*^−/−^ mice reconstituted with WT hematopoietic cells (WT > *Stat1*^−/−^), comparably displayed intermediate susceptibility (Figure 6C). Collectively, IFN-γ-STAT1 signaling in both the Kupffer and sinusoidal endothelial cells equally contributed to the liver injury via TF induction (Figure 6D).

#### 3.1.3. Con A, IFN-γ, and TNF Are Necessary and Sufficient for Massive Liver Injury

**Figure 7.**
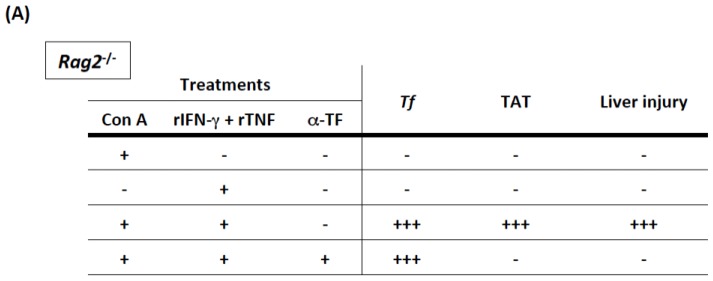

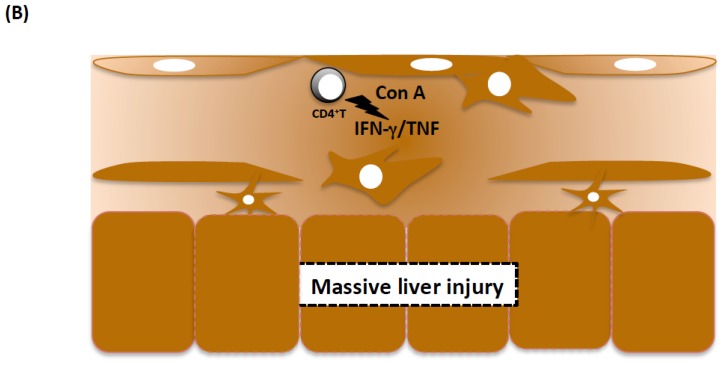
TNF/IFN-γ can replace T-cell function. (**A**) *Rag2*^−/−^ mice lack T cells that can be activated by Con A. *Rag2*^−/−^ mice did not show the hepatic induction of *Tf*, increase in plasma levels of TAT, or liver injury after Con A challenge. Unlike in WT mice, *Rag2*^−/−^ mice did not display the hepatic induction of *Ifnγ* or *Tnf*, the cytokines triggering the prothrombotic response as shown in the text. This suggests that T cells might be a major cell source of IFN-γ and TNF. Indeed, supplementation with recombinant IFN-γ (rIFN-γ) and rTNF rendered *Rag2*^−/−^ mice highly susceptible to Con A challenge, in terms of hepatic induction of *Tf*, elevation of plasma levels of TAT, and liver injury. Notably, the neutralization of TF with α-TF antibody protected against this thrombotic liver injury as well. Intriguingly, challenge with rIFN-γ and rTNF alone did not induce any of these changes; and (**B**) A proposed model for Con A-induced liver injury. Upon Con A challenge, T cells, particularly CD4^+^, produce IFN-γ and TNF. The IFN-γ-STAT1 pathway, TNF signaling, and Con A signaling induce TF expression equally in the sinusoidal endothelial and Kupffer cells. The TF expressed in those cells activates the coagulation cascade within the sinusoid, which eventually results in sinusoidal thrombosis, leading to the development of liver injury with endothelial damage. This subsequently allows the hepatocytes and stellate cells, which are segregated from the sinusoid under normal conditions, to come in contact with circulating coagulation factors. Con A treatment also induces TF expression in these two types of cells. Collectively, the combined expression of TF might participate in the acceleration of massive liver injury during sinusoidal rupture.

*Rag2*^−/−^ mice that lack T and B cells are resistant to Con A-induced hepatitis (Figure 7A), accompanied by the lack of hepatic induction of IFN-γ and TNF. This suggests that T cells might serve as IFN-γ/TNF-producing cells. In fact, simultaneous injection of rIFN-γ and rTNF render *Rag2*^−/−^ mice susceptible to Con A treatment in terms of hepatic *Tf* induction and development of massive liver injury (Figure 7A). Furthermore, neutralization of TF completely rescued *Rag2*^−/−^ mice from the IFN-γ/TNF/Con A-induced liver alterations (Figure 7A).

All the results thus far suggest the following: upon Con A challenge, T cells promptly produce IFN-γ and TNF. In turn, Con A, IFN-γ, and TNF signaling combine to induce TF expression in Kupffer and endothelial cells. The Con A-induced signaling pathway involved in this mechanism has yet to be elucidated. These events hyperactivate the procoagulant pathway to produce intrasinusoidal microthrombi. In addition, both hepatocytes and stellate cells exhibit enhanced TF expression following Con A challenge [55]. These cells are segregated from the sinusoid under normal conditions (Figure 1). However, sinusoidal damage brings even those “hidden” cells in contact with circulating coagulation factors to participate in the further activation of the coagulation cascade. Subsequently, these events result in massive liver injury (Figure 7B). Recently, heparin pretreatment was reported to partly protect against FasL-induced lethal liver injury, presumably via inhibition of intravascular coagulation [39,61]. From these observations and ours, we propose that intrasinusoidal microthrombi with multifoci may contribute to prompt and burst development of liver necrosis in patients with massive liver injury such as that in acute liver failure and/or acute-on-chronic liver failure.

### 3.2. APAP-Induced Acute Liver Injury

APAP is a commonly used nonprescription analgesic and antipyretic in many developed countries, including Japan. Accidental or intentional overdose with APAP results in lethal liver injury with hypercoagulability [62]. The APAP metabolite is directly toxic to hepatocytes [63]. Recently, inflammatory and hypercoagulability responses following the initial hepatocyte damage induced by the APAP metabolite were demonstrated to be critical for the lethal exacerbation of APAP-induced liver injury.

#### 3.2.1. Hypercoagulability in APAP-Induced Hepatotoxicity

Administration of a toxic dose of APAP induced lethal hepatitis in mice. It is well established that APAP induces hepatic inflammation and liver cell damage [64]. The mechanism of the development of APAP-induced hepatic inflammation was unclear. However, recently, the following mechanism was clearly demonstrated. APAP treatment induces hepatocyte cell death by the direct action of the APAP metabolite, resulting in liberation of dead cell-derived free DNA. This DNA in turn induces inflammatory responses that produce precursor IL-1β and IL-18 in sinusoidal endothelial cells via TLR9 activation. In parallel, the NLRP3 inflammasome is activated and then it secretes mature IL-18 and IL-1β, although the agonist(s) involved in the NLRP3 inflammasome activation is unknown. *Il18*^−/−^ mice are somewhat resistant to APAP-induced mortality [64]. Treatment with neutralizing IL-1β antibody or TLR9 antagonists significantly rescues mice from APAP-induced lethality [64]. Thus, APAP induces hepatic inflammation via the activation of innate signal receptors sensing extracellular and intracellular molecules in host cells. In addition to hepatic inflammation, coagulation disorder has been demonstrated to be involved in APAP-induced liver injuries [65]. After APAP challenge, mice exhibit substantial fibrin deposition in their livers. Plasma TAT levels were elevated, followed by an increase in plasma PAI-1 levels, suggesting both local and systemic hypercoagulability. Heparin treatment protected against liver injury. Furthermore, *Tf*^−/−^*TF* transgenic mice, cells of which constitutively express fixed low levels of human TF but lack the capability to express endogenous murine TF, are resistant to APAP-induced liver injury [65]. More recently, hepatocytes were reported to be a major cellular source of TF in the liver [66]. They reported that primary cultured murine hepatocytes expressed TF on their surface even under normal conditions. Furthermore, hepatocytes isolated from WT mice have the potential to exert procoagulant activity *in vitro*. In contrast, hepatocytes isolated from liver-specific TF conditional knockout mice lack this capacity. Notably, intravenous injection of normal hepatocytes, but not those from the hepatocyte-specific TF-deficient mice, induced plasma TAT elevation [66]. Thus, hepatocytes themselves are capable of activating the coagulation cascade by expressing TF. Intriguingly, the mutant mice developed liver injury in the limited early phase after APAP treatment, as did the WT mice. This is perhaps due to the direct, hepatocytocidal action of the APAP-derived toxic metabolite. Therefore, it is plausible that during liver damage induced by the APAP-derived hepatocytotoxin, the injured hepatocytes might trigger the activation of the procoagulation system by contact with the procoagulant factors in the circulation. Under normal conditions, however, hepatocytes cannot activate the coagulation cascade because of their localization apart from the sinusoid (Figure 1). From this, we may assume that under poor anticoagulable conditions, liver damage by itself has a predisposition to trigger hypercoagulability irrespective of the mechanism by which liver damage is induced.

#### 3.2.2. Kupffer Cells Play a Beneficial Role

As mentioned previously, Kupffer cells play a critical role in the *P. acnes*-induction of LPS sensitization and Con A-induced severe hepatitis by activating hepatic granuloma formation and inducing TF expression, respectively (Figures 2 and 6). In contrast to those models of acute liver injuries, Kupffer cells have been reported to negatively regulate APAP-induced acute liver injury. The liver injury in Kupffer cell-ablated mice was more severe than that in Kupffer cell-sufficient control mice [67–69]. Kupffer cells play several protective roles. After APAP challenge, various anti-inflammatory cytokines such as IL-4, IL-13, and IL-10 are produced, as well as proinflammatory cytokines such as IL-1β and IL-18 [64,70]. Kupffer cell-ablated mice displayed impaired induction of IL-10 and IL-18-binding protein (BP) following APAP treatment [67]. As the IL-18 BP serves as a natural antagonist of IL-18 with a higher affinity for IL-18 than the affinity of IL-18 receptor (IL-18R) for IL-18 [71], reduction of IL-18 binding protein (IL-18 BP) induction might conceivably result in the development of more severe liver injury [64]. *Il10*^−/−^ mice are highly susceptible to APAP-induced liver injury [70], suggesting that Kupffer cells might negatively regulate inflammation by producing IL-10 as well. In addition to the production of anti-inflammatory molecules, Kupffer cells are capable of repairing liver injury. Kupffer cells isolated from APAP-treated mice expressed significantly higher levels of angiogenesis-associated molecules than those from untreated mice. In fact, upon APAP treatment, Kupffer cell-ablated mice showed prolonged vascular leakage in their livers when compared with Kupffer cell-sufficient normal mice [68,69]. Thus, Kupffer cells play a beneficial role in APAP-induced liver injury by producing anti-inflammatory molecules and pro-angiogenic factors.

From the previous reports, we may assume the following scenario for APAP-induced liver injury: the APAP metabolite rapidly and directly induces hepatocyte cell death, followed by the induction of inflammatory responses via the activation of TLR9 and the NLRP3 inflammasome. Dead hepatocytes and the resultant hepatic inflammation might disrupt the sinusoidal structure, eventually resulting in the exposure of TF-expressing hepatocytes to the blood flow, leading to the production of intrasinusoidal microthrombi. Kupffer cells negatively regulate liver injury by producing anti-inflammatory cytokines and repair-initiating pro-angiogenic molecules. Collectively, these events might converge on the development of acute liver failure.

## 4. Conclusions

Kupffer cells show pathological and beneficial actions in the context of different pathogenic processes via the activation of the different machineries they possess. Recent studies have revealed a complicated network among these various systems, such as innate/acquired immunities, metabolic pathways, wound-healing mechanisms, and procoagulant/anticoagulant systems. These systems were previously believed to be regulated independently from one another. We are now beginning to unravel the complicated relationships among them. In the near future, we will have developed the tools to prevent and/or treat intractable liver diseases that may result from these complicated networks involving multiple biological systems.
